# Supplementary material for: Carbon dots functionalized papers for high-throughput sensing of 4-chloroethcathinone and its analogues in crime sites
Source: R Soc Open Sci. 2019 Sep 11;6(9):191017. doi: 10.1098/rsos.191017 (PMC6774952; doi:10.1098/rsos.191017)
Supplement: Supplementary Information [file rsos191017supp1.docx]

**Supplementary information**

Carbon Dots Functionalized Papers for High-Throughput Sensing of 4-Chloroethcathinone and its analogues in Crime Sites ^†^

Yao-Te Yen^1,2^, Yu-Syuan Lin^1^, Ting-Yueh Chen^2^, San-Chong Chyueh^2^, and Huan-Tsung Chang ^2,3^*

^1^Department of Chemistry, National Taiwan University, Taipei 10617, Republic of China (Taiwan)

^2^Department of Forensic Science, Investigation Bureau, Ministry of Justice, Xindian Dist, New Taipei City 23149, Republic of China (Taiwan)

^3^Department of Chemistry, Chung Yuan Christian University, Chungli District, Taoyuan City 32023, Republic of China (Taiwan)

**Correspondence:** Prof. Huan-Tsung Chang, Department of Chemistry, National Taiwan University, 1, Section 4, Roosevelt Road, Taipei 10617, Taiwan; E-mail: [changht@ntu.edu.tw](mailto:changht@ntu.edu.tw); Tel: 011-886-2-3366-1171

**Keywords:** sensor, carbon dots, abuse drugs, cathinones, cocaine.


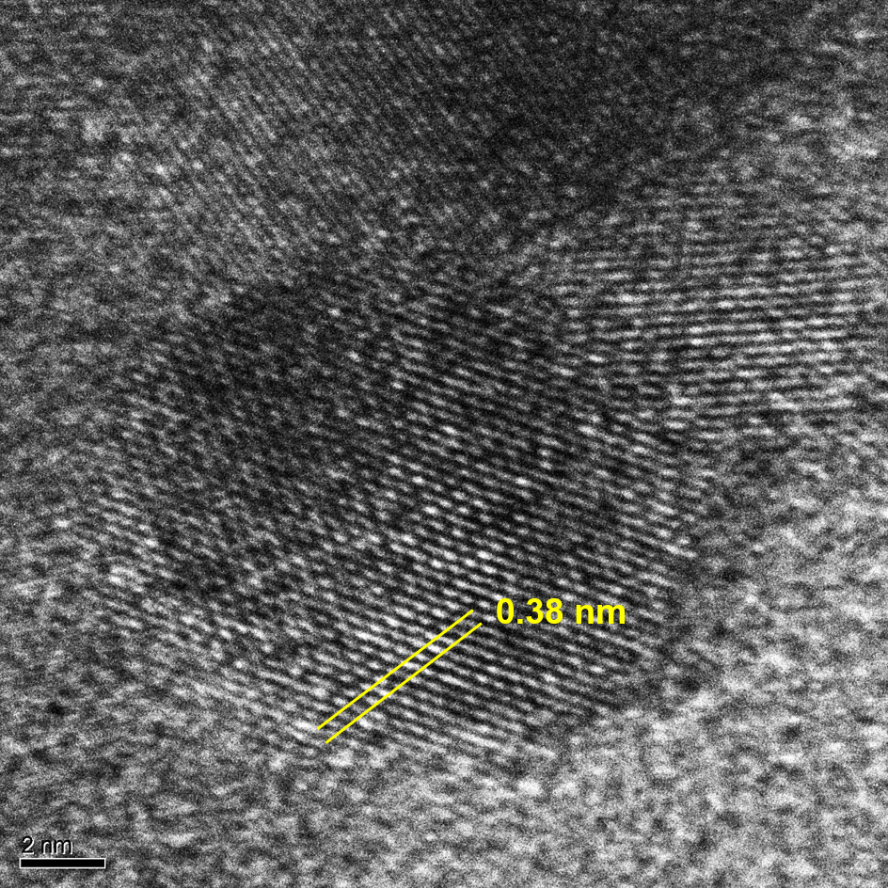


**Figure S1**. HRTEM image of C-dots.


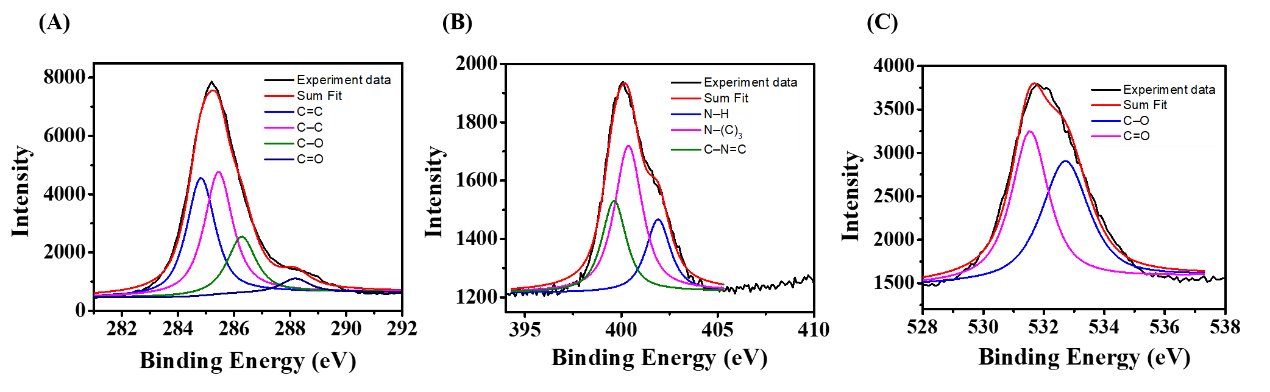


**Figure S2**. Deconvoluted (A) C1s, (B) N1s, and (C) O1s XPS spectra of C-dots.


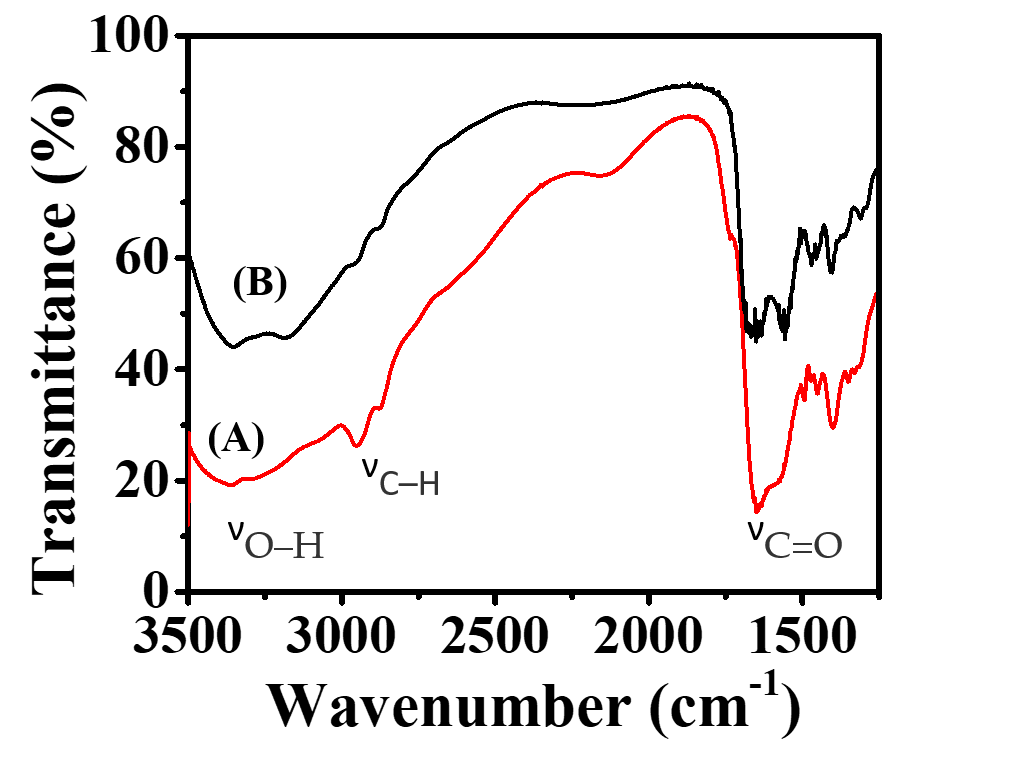


**Figure S3.** FTIR spectra of (A) the C-dots and (B) arginine.


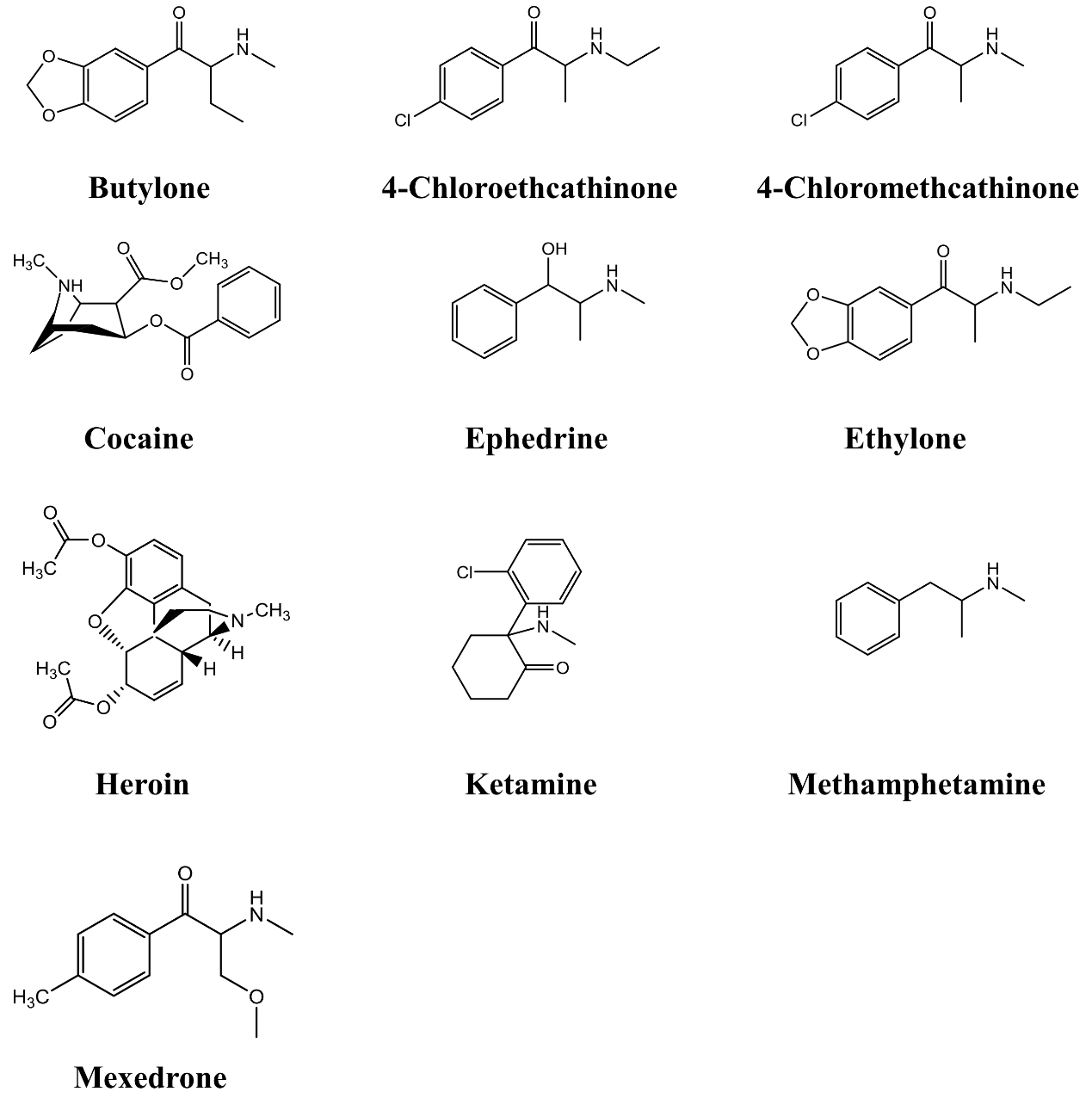


**Figure S4**. Structures for some tested abused drugs.


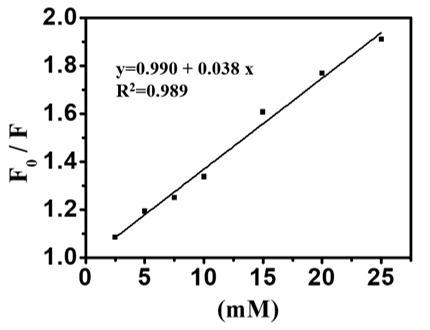


**Figure S5**. Stern-Volmer plot of C-dots in sodium phosphate buffer (90 mM, pH 11.0) for 4-chloroethcathinone. Stern-Volmer equation: F_0_ / F = 1 + K_SV_ [Q], where F_0_ and F are the PL intensities of C-dots in the absence and presence of 4-chloroethcathinone, respectively, [Q] is the concentration of 4-chloroethcathinone, and K_SV_ is the quenching constant for 4-chloroethcathinone.


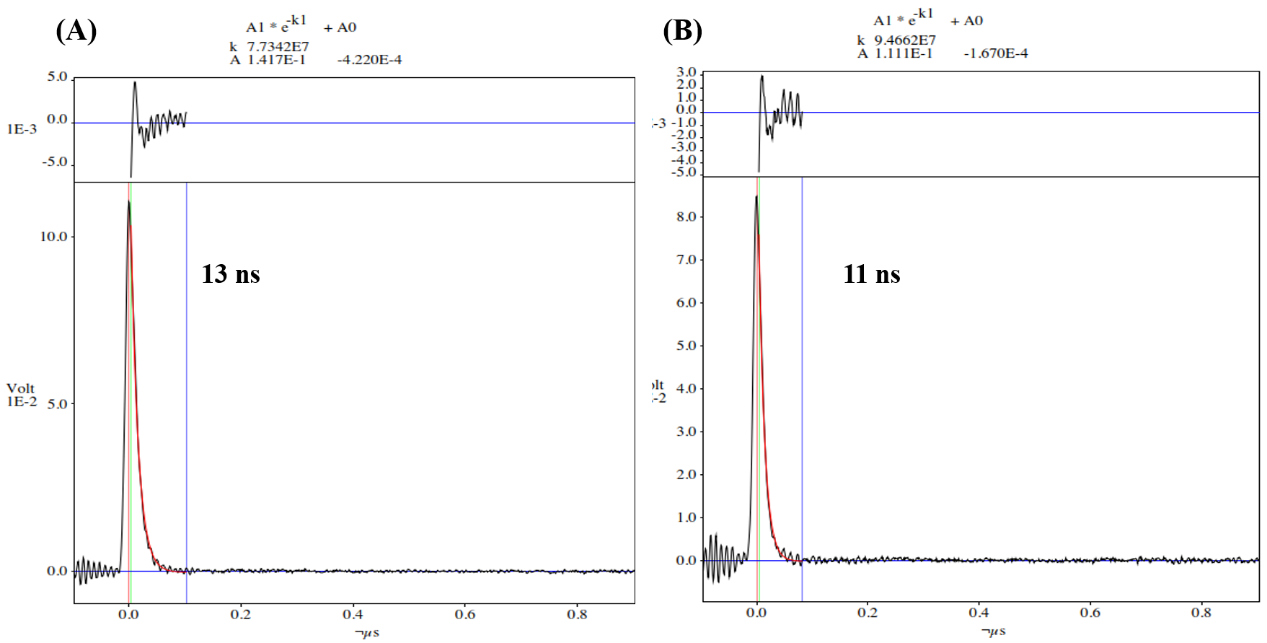


**Figure S6.** Fluorescence lifetime decay curves of C-dots (A) without and (B) with containing 4-chloroethcathine (15 mM) at emission/excitation wavelength of 430/360 nm.


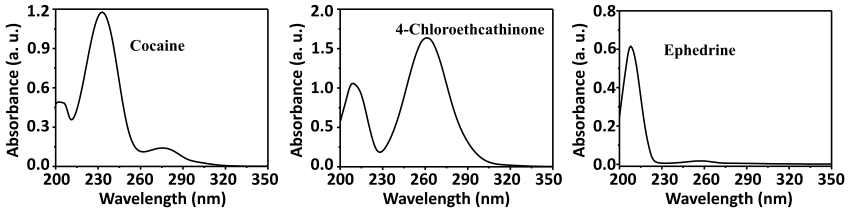


**Figure S7**. Absorption spectra of cocaine, 4-chloroethcathinone, and ephedrine.
